# Tunable light filtering by a Bragg mirror/heavily doped semiconducting nanocrystal composite

**DOI:** 10.3762/bjnano.6.18

**Published:** 2015-01-16

**Authors:** Ilka Kriegel, Francesco Scotognella

**Affiliations:** 1 Dipartimento di Fisica, Politecnico di Milano, Piazza Leonardo da Vinci 32, 20133 Milano, Italy; 2 Center for Nano Science and Technology@PoliMi, Istituto Italiano di Tecnologia, Via Giovanni Pascoli 70/3, 20133 Milano, Italy

**Keywords:** active optical component, electronic band gap nanostructure, localized surface plasmon resonance, photonic crystal

## Abstract

Tunable light filters are critical components for many optical applications in which light in-coupling, out-coupling or rejection is crucial, such as lasing, sensing, photovoltaics and information and communication technology. For this purpose, Bragg mirrors (band-pass filters with high reflectivity) represent good candidates. However, their optical characteristics are determined during the fabrication stage. Heavily doped semiconductor nanocrystals (NCs), on the other hand, deliver a high degree of optical tunability through the active modulation of their carrier density, ultimately influencing their plasmonic absorption properties. Here, we propose the design of an actively tunable light filter composed of a Bragg mirror and a layer of plasmonic semiconductor NCs. We demonstrate that the filtering properties of the coupled device can be tuned to cover a wide range of frequencies from the visible to the near infrared (vis–NIR) spectral region when employing varying carrier densities. As the tunable component, we implemented a dispersion of copper selenide (Cu_2−x_Se) NCs and a film of indium tin oxide (ITO) NCs, which are known to show optical tunablility with chemical or electrochemical treatments. We utilized the Mie theory to describe the carrier-dependent plasmonic properties of the Cu_2−x_ Se NC dispersion and the effective medium theory to describe the optical characteristics of the ITO film. The transmission properties of the Bragg mirror have been modelled with the transfer matrix method. We foresee ease of experimental realization of the coupled device, where filtering modulation is achieved upon chemical and electrochemical post-fabrication treatment of the heavily doped semiconductor NC component, eventually resulting in tunable transmission properties of the coupled device.

## Introduction

Optical filters are fundamental components employed in almost all optical setups and devices. For example, they play a very important role in microfluidic devices, which are very interesting for point-of-care diagnostics [1–2]. Very interesting strategies to fabricate colour filters, without auto-fluorescence, are proposed in the literature [3]. However, in many applications, such as in microfluidic devices, where the detection of more than one analyte is often necessary, the use of tunable filters would be a great advantage.

A very efficient class of filters are Bragg mirrors, also called one-dimensional photonic crystals [4–6]. Bragg mirrors can be made of dense materials [7], but also with mesoporous materials or nanoparticles [8–10]. Their use has been exploited in several types of devices, such as distributed feedback lasers [11–15], sensors [16–17], absorption enhancement for photovoltaics [18] or in dye-sensitized solar cells [19–21]. Furthermore, nanoparticle-based photonic crystals have been employed for switching applications [22–25]. An advantage of Bragg mirrors is that a proper design enables access to a variety of different wavelength regions, determined by the design of the respective photonic structure [4–6]. Parameters that can be varied are layer thickness, material refractive index and stacking sequence. However, the tunability is limited during the production stage.

On a different front, heavily doped semiconductor NCs have been receiving increasing attention in recent years. Their high level of doping leads to localized surface plasmon resonances (LSPRs) mostly located in the vis–NIR [26]. The advantage of doped semiconductor nanostructures is the option to chemically and electrochemically modify their plasmon resonance frequencies by changing the material’s carrier density. For copper chalcogenide NCs, chemical manipulation has been demonstrated in response to oxidizing and reducing treatments [27–32]. The exposure of a solution of copper chalcogenide NCs, such as Cu_2−x_S or Cu_2−x_Se, to oxygen or the addition of an oxidizing agent leads to a blue shift and increase in intensity of the NIR LSPR. Notably, this process can be entirely reversed through the addition of reducing agents, completing the full cycle of tunability [29,32]. In metal oxide NCs a dynamic modulation of the LSPR has been demonstrated through a fully reversible electrochemical treatment. In this approach, the change in plasmon absorption is achieved through electrochemical doping, activated by applying a voltage to conducting films of metal oxide NCs [33]. In a recent study, the modulation of transmittance was electrochemically achieved in layers of transparent conducting NCs, such as ITO [34–36]. Moreover, plasmonic effects have been exploited for electrochromic applications for the direct modulation of the transmittance of solar energy [34–37].

In this study, we propose a tunable filter based on photonic crystals (Bragg mirrors) coupled to heavily doped semiconductor NCs with plasmonic absorption properties. We report the modelling of a Bragg mirror coupled to a dispersion of chemically tunable Cu_2−x_Se NCs and electrochemically switchable NC films of ITO. In both cases switching is based on a modulation of their carrier density and, thus, this combination will ultimately result in tunable, broadband light transmission. We model the structure by implementing the transfer matrix method to describe the optical properties of the Bragg mirror. The Mie theory describes the tunable plasmonic properties of the Cu_2−x_Se NCs, and the effective medium theory is employed to describe the tunable optical characteristics of the ITO film. Since the plasmon peak of the NCs can be dynamically modified, we envisage a filter with properties that can be fine-tuned through a broad range of frequencies according to the desired application.

## Theoretical Methods

To calculate the transmission spectra of the photonic crystal component in the structure, the transfer matrix method was employed, which is a general technique widely used in optics for the description of stacked layers and extensively described in [38]. Isotropic, nonmagnetic materials in a layered structure on a glass substrate, followed by the multilayer in air, have been considered. The incidence of the light has been considered perpendicular to the multilayer surface. *n*_0_ and *n*_S_ are the refractive indexes of air and glass, respectively, and *E*_m_ and *H*_m_ are the electric and magnetic fields in the glass substrate, respectively. The electric and magnetic fields in air, *E*_0_ and *H*_0_, have been obtained by solving the following system:

[1]
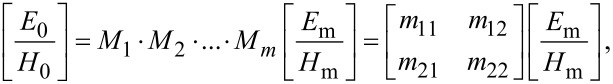


where


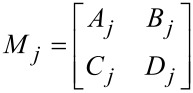


and *j* = (1, 2, …, *m*) is the characteristic matrix of each layer. The elements of the transmission matrix *ABCD* are

[2]
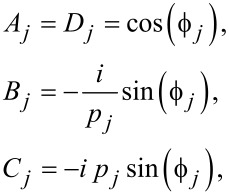


where *n_j_* and *d_j_*, contained in the angle 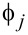, are the effective refractive index and the thickness of the layer *j*, respectively. In the case of normal incidence of the probe beam, the phase variation of the wave passing the *j*-fold layer is 
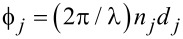
, while the coefficient 
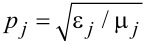
 in the transverse electric wave and *q_j_* = 1/*p_j_* replaces *p_j_* in the transverse magnetic wave. Inserting Equation 3 into Equation 4 and using the definition of the transmission coefficient

[4]
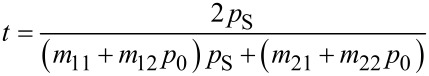


it is possible to write the light transmission as

[3]
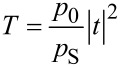


where *p*_S_ represents the substrate and *p*_0_ air. To calculate the optical properties of the doped Cu_2−x_Se NC dispersion in the region of plasmonic absorption we used the quasi-static approximation of Mie scattering theory, which provides the following expression for the absorption cross-section σ*_A_*:

[5]
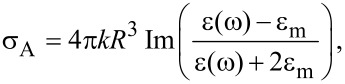


where ε(ω) is the bulk dielectric function at the optical frequency ω and ε_m_ is the dielectric constant of the surrounding medium. The optical extinction of ultra-small particles is dominated by absorption, but a first order correction to the quasi-static approximation should be included to account for a scattering contribution, with a scattering cross-section given by the following expression:

[6]
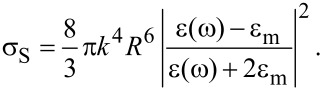


In the above equations, *k* = *n*_m_ω/*c* with *n*_m_ = (ε_m_)^1/2^ is the refractive index of the dielectric environment and *c* is the speed of light in vacuum [39]. The Drude model has been assumed to account for the optical properties in heavily doped semiconductor NCs in the NIR, with a complex dielectric function given by [27–31]:

[7]
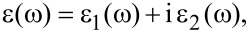


where

[8]
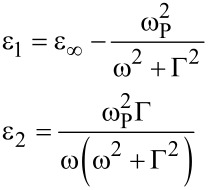


where Γ is the free carrier damping and

[9]
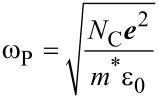


is the plasma frequency of the free carriers of the system. Here, *N*_C_ is the carrier density, ***e*** the charge of the electron, m* the effective mass and ε_0_ the vacuum dielectric permittivity.

The absorption of the ITO NC film has been described by applying the effective medium theory or Maxwell–Garnett effective medium approximation (MG-EMA) [40–42]. This theory has been developed to describe the macroscopic properties of a composite material and to average the medium dielectric function according to the multiple values of the constituents of the composite material. The effective dielectric function (ε_eff_) of a film of ITO NCs can be described by the MG-EMA as follows:

[10]
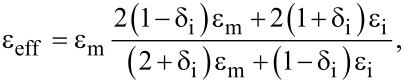


where ε_m_ is the medium dielectric constant, ε_i_ is the frequency-dependent dielectric function of the bulk material (in this case approximated by the Drude model, Equations 8 and 9) and δ_i_ accounts for the volume fraction. Within this theory only far field interactions are taken into account, while near field interaction among the NCs are neglected. The absorbance of the NC films is finally calculated from the imaginary part of the dielectric function [(ε_eff_)^1/2^] and the Beer–Lambert law [42].

## Results and Discussion

**Figure 1:**
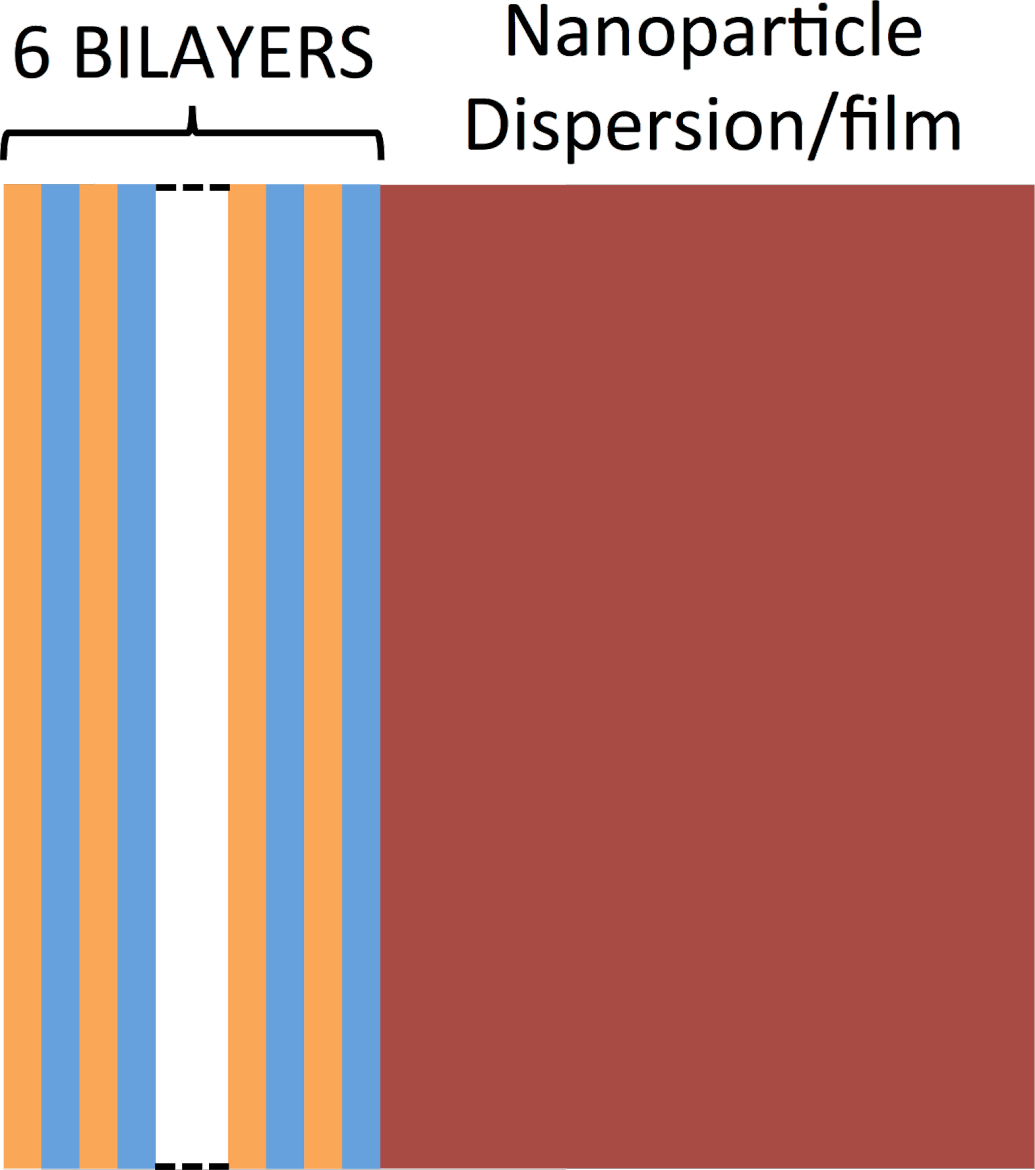
Scheme of the device in which a Bragg mirror (photonic crystal), illustrated by alternating orange and blue layers, is coupled to a dispersion of Cu_2−x_Se NCs in toluene or an ITO NC film (dark red) to act as a tunable light filter. In the presented device, tunability is envisaged upon the modulation of the carrier density in the heavily doped semiconductor NC component through chemical and electrochemical treatments, ultimately leading to tunable plasmonic light absorption.

Figure 1 provides a schematic of the proposed device, a Bragg mirror (blue and orange layers in Figure 1) coupled to either a dispersion of Cu_2−x_Se NCs or an ITO NC film, sketched in dark red.

**Figure 2:**
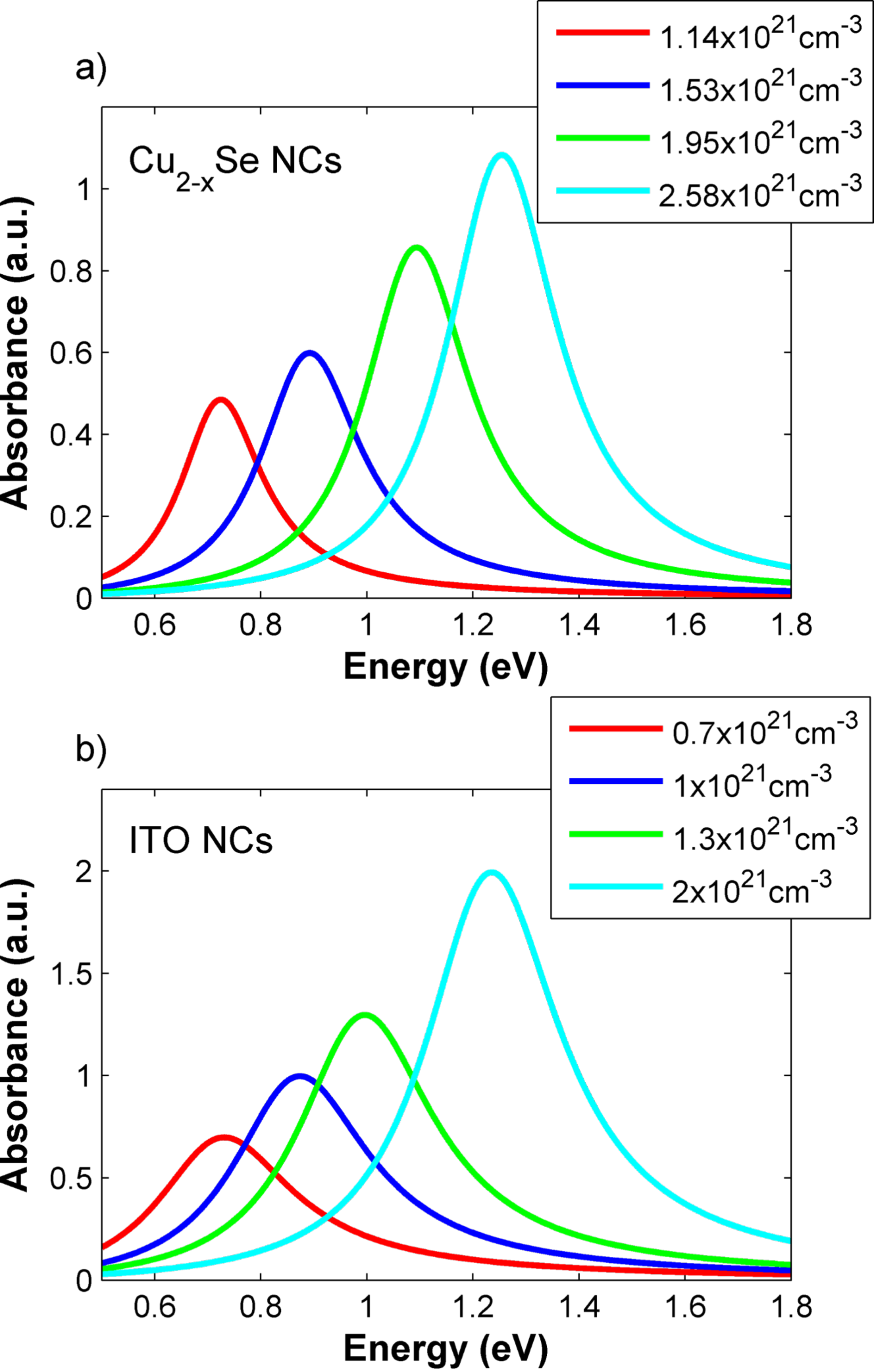
Illustration of the tunable optical properties of a) a Cu_2−x_Se NC dispersion and b) an ITO NC film as a function of carrier density *N*_C_ = 1.14, 1.53, 1.95 and 2.58 × 10^21^ cm^−3^ (Cu_2−x_Se NCs) and *N*_C_ = 0.7, 1, 1.3 and 2 × 10^21^ cm^−3^ (ITO NCs). The given absorption spectra are calculated according to a) Mie theory and b) the MG-EMA assuming a Drude-like behaviour of the free carriers in the system.

In the following, we demonstrate the modelling of the transmission properties of the proposed device. We first consider the plasmonic absorption properties of the heavily doped NC component and demonstrate its modulated absorption properties through an appropriate choice of carrier densities in the structure. Thereafter, we demonstrate how the absorption of the Bragg mirror is altered when coupled to a layer of NCs with varying carrier densities, and finally present the transmission properties of the actual device. Concerning the Cu_2−x_Se NCs, we have considered a dispersion of Cu_2−x_Se NCs in toluene with a spherical shape and a diameter of 15 nm, with ε_∞_ = 11 [27,43]. The carrier density-dependent effective mass and damping constant were taken from [43] with the following parameters: *m*_1_* = 0.445·*m*_0_, *m*_2_* = 0.394·*m*_0_, *m*_3_* = 0.334·*m*_0_, and *m*_4_* = 0.336·*m*_0_ and Γ_1_ = 0.189 eV, Γ_2_ = 0.232 eV, Γ_3_ = 0.244 eV, and Γ_4_ = 0.254 eV for carrier densities of 1.14, 1.53, 1.95 and 2.58 × 10^21^ cm^−3^, respectively. The dispersion is in toluene, such that ε_m_ = 2.24. The concentration of the NCs in the dispersion was set to 1 × 10^17^ cm^−3^, and the dispersion thickness to 1 mm. It was demonstrated that by oxidation or reduction of the NCs, the carrier density can be tuned, resulting in the variation of the plasma frequency. Figure 2a shows the calculated absorption spectra of Cu_2−x_Se NCs for the different carrier densities (as given above) and the corresponding parameters of effective mass and damping constant. A blue shift of up to 0.7 eV and an increase in intensity with increasing carrier density is observed. The calculated results are in good agreement with experimental results obtained in [27–32]. In those works, a modulation of the plasmonic absorption was triggered through the addition of chemical agents, inducing oxidation and reduction. This in turn leads to a variation of the carrier density and a blue shift (for oxidation) or red shift (for reduction) of the plasmon resonance over a wide range of frequencies. Such observation underlines that our model appropriately describes the absorption properties of a Cu_2−x_Se NC dispersion, where a tuning of the plasmon resonance can be triggered by chemical means.

As a second example we have considered a film of ITO NCs with a thickness of 5 µm employing the MG-EMA to calculate its absorption properties [28]. The refractive index of the film is 1.5 (same as that of the glass). For the NCs we consider a free carrier damping Γ = 0.31 eV and a high frequency dielectric permittivity of ε_∞_ = 4 [28–30]. The volume fraction of ITO in the film is chosen to be 0.01 [35]. In Figure 2b the absorption spectra of the ITO film with different carrier densities, namely, 0.7, 1, 1.3 and 2 × 10^21^ cm^−3^ are depicted. This theoretical approach has been successfully employed in a recent work to describe the absorption properties of ITO NC films [37,42]. Experimentally, the plasmon frequency of ITO NC films was reversibly tuned by electrochemical post-treatment leading to a switching of the plasma frequency throughout the NIR spectral region [33,36–37]. Taken altogether, the proposed theoretical framework is appropriate to describe the tuning of light absorption with doped semiconductor NCs in a dispersion or in films, both thoroughly confirmed in experimental and theoretical works [29,32–33,36–37].

**Figure 3:**
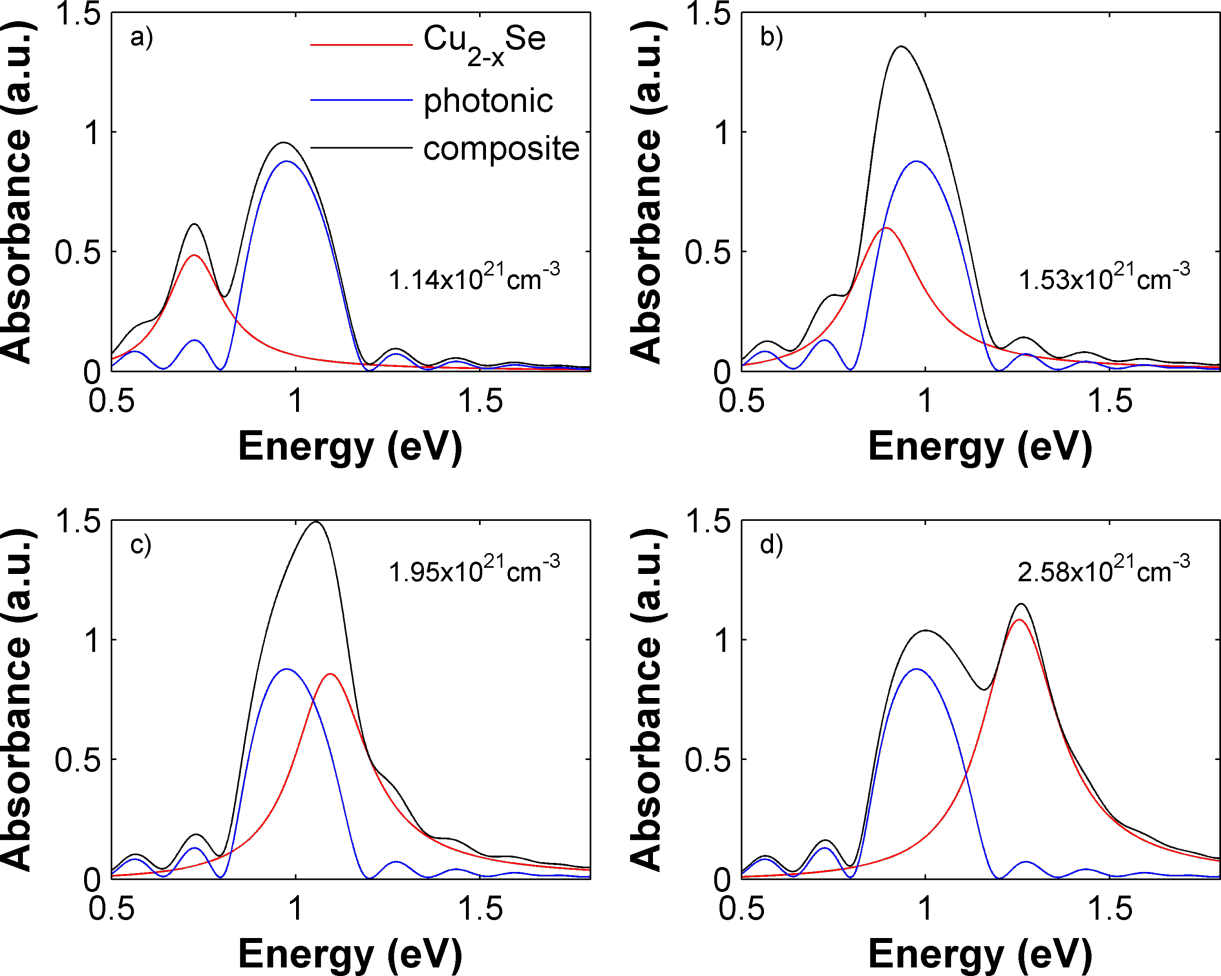
Absorption properties of a Bragg mirror (blue curves) coupled to a dispersion of Cu_2−x_Se NCs (red curves) for different carrier concentrations of a) 1.14, b) 1.53, c) 1.95 and d) 2.58 × 10^23^ cm^−3^. The black curves illustrate the optical properties of the coupled device.

To describe the optical characteristics of the Bragg mirror, we have designed an alternating stack of six bilayers of silicon dioxide (SiO_2_, *n* = 1.46) and zinc oxide (ZnO, *n* = 2) with a layer thickness of 180 nm. The refractive index dispersion for these two metal oxides was not considered since they are approximately constant in the energy range considered in this study. The absorption spectrum of the multilayer shows a peak centred at 1 eV (blue curves in Figure 3), due to the occurrence of the photonic band gap. The chosen materials, layer thicknesses, and number of layers are typical variable parameters for Bragg mirrors. The absorption properties of such variable stackings are well-studied and demonstrated in experimental frameworks, thus, appropriate for the actual applicability of our proposed device [7,44].

By coupling the engineered Bragg mirror with the Cu_2−x_Se NC dispersion, we obtain a composite device showing an absorption spectrum that is the sum of the photonic band gap and the plasmon absorption (Figure 3). In Figure 3 the blue curve depicts the absorption spectrum of the Bragg mirror alone, characterized by the photonic band gap at around 1 eV. The red curves describe the absorption of the Cu_2−x_Se NC dispersion for different carrier densities, namely, 1.14, 1.53, 1.95 and 2.58 × 10^23^ cm^−3^ for Figure 3a–d, respectively, and as calculated above (Figure 2). The black curve describes the overall absorption of the Bragg mirror/NC composite. Through the appropriate choice of materials (i.e., Cu_2−x_Se NC dispersion), and the clever design of the Bragg mirror, the absorption properties of the coupled device result in an extension of the absorption to either the red, for low carrier densities of the heavily doped semiconductor NCs, or the blue for high carrier densities. This combination enables a filtering tunability, which is not achievable for the Bragg mirror alone.

**Figure 4:**
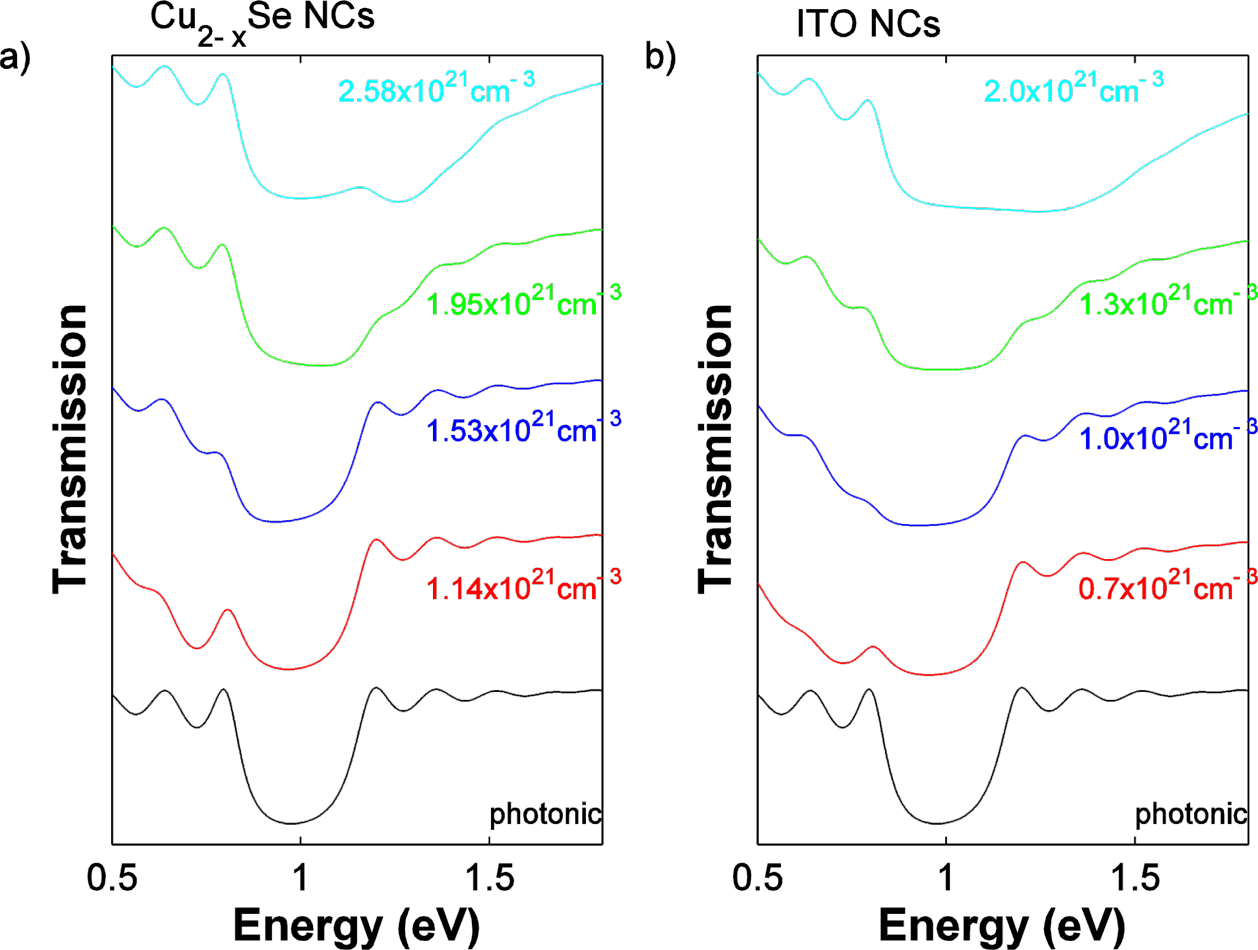
Transmission of the tunable light filter for a Bragg mirror coupled to a) a Cu_2−x_Se NCs dispersion and b) to an ITO NC film with varying carrier concentrations representing the band gap tuning of the Bragg mirror. The black curves correspond to the bare Bragg mirror. In both presented devices, the filtering covers a broader range which is blue shifted (for higher carrier concentrations) and red shifted (for lower concentrations), representing the tunability of the filter upon carrier density modulation in the heavily doped semiconductor NC component.

The overall transmission properties of the coupled device are demonstrated in Figure 4, which actually demonstrates its active filtering modulation. Shown here is the transmission spectrum for the coupling of the Bragg mirror to the Cu_2−x_Se NC dispersion (Figure 4a) and to the ITO NC films (Figure 4b). We clearly observe that (with respect to the photonic crystal alone) the composite shows a larger band. Furthermore, by changing the carrier density in the Cu_2−x_Se NC dispersion and the ITO NC films from 1.14, to 1.53, 1.95 and finally to 2.58 × 10^21^ cm^−3^, we observe a coverage of a broader absorption range from the red to the blue part of the visible spectrum. The overall transmission spectrum of the composite is therefore reversibly tunable, due to the possibility of the dynamic and reversible carrier density of the system, inducing a blue or red shift of the plasmonic absorption in the heavily doped NC component. Moreover, by changing the refractive index ratio of the Bragg mirror, the linewidth of its photonic band gap can be varied, allowing yet another degree of freedom in the design of the proposed coupled device (see Supporting Information File 1, Figure S1).

The unique combination of a highly reflecting Bragg mirror with strongly absorbing NCs and the resulting exceptional tuning properties allows for a filtering device that delivers the outstanding property of active transmission modulation over a broad range of frequencies. We want to point out that we envisage the band gap tuning of the Bragg mirror/NC composite as demonstrated in Figure 4 to be experimentally feasible upon a fully reversible post-fabrication treatment. Once the composite is fabricated by coupling the NC dispersion or film to the Bragg mirror, tuning of the transmission is realized by applying chemical or electrochemical treatments, while keeping the concentration and thickness of the dispersions or films and the Bragg mirror intact. The wavelength tuning is realized solely by influencing the carrier density of the NCs, which in turn leads to the altered absorption properties and the shift through a broad wavelength range, as demonstrated in Figure 4. We further highlight that the transmission modulation in an actual device would not occur in steps, as demonstrated by the choice of discrete given carrier densities, but would rather change continuously covering the entire range of wavelengths. Moreover, the use of a photonic crystal and of a heavily doped semiconductor NC component provide filtering without auto-fluorescence (a detailed discussion of auto-fluorescence: [3,44]. For this reason, the proposed filter is particularly appropriate for sensor devices, but also for biological applications, where low fluorescence signals are detected.

## Conclusion

We introduced the design and theoretical framework of a non-emissive, actively tunable filter composed of a Bragg mirror coupled to a thin layer of heavily doped, plasmonic semiconductor NCs. The high reflectivity of the Bragg mirror and the strong extinction coefficient of the heavily doped semiconductor NCs, combined with the exceptional tuning properties of the plasmonic absorption, results in an effective filtering modulation. We demonstrated that the band gap tuning over a wide range of frequencies can be achieved when the carrier density of the plasmonic NC layer is properly designed. Within this work, we envisaged a Cu_2−x_Se NC dispersion or an ITO NC film as tunable components. In both systems, the experimental modulation of their absorption properties by chemical or electrochemical means has been shown in a number of recent works demonstrating their actual applicability [27–34]. In this work, the calculations carefully take into account all the physical parameters considered to model the aforementioned experimental data. However, we envisage the use of any other type of highly doped semiconductor NC, allowing for a clever design of the filtering properties. We foresee a straightforward realization of such filters by coupling a NC dispersion or film with a Bragg mirror, deposited with spin coating or sputtering techniques [7–8,45]. The application of this kind of tunable filtering component is very promising for various optical applications such as lasing, sensing, photovoltaics or information and communication technology.

## Supporting Information

File 1 Transmission dependence on refractive index of the tunable filter for a Bragg mirror coupled to an ITO NC film.
