# Supplementary material for: Tunable light filtering by a Bragg mirror/heavily doped semiconducting nanocrystal composite
Source: Beilstein J Nanotechnol. 2015 Jan 16;6:193–200. doi: 10.3762/bjnano.6.18 (PMC4311676; doi:10.3762/bjnano.6.18)
Supplement: File 1 — Transmission dependence on refractive index of the tunable filter for a Bragg mirror coupled to an ITO NC film. [file Beilstein_J_Nanotechnol-06-193-s001.pdf]

Supporting Information

for

Tunable light filtering by a Bragg mirror/heavily doped semiconducting nanocrystal composite

Ilka Kriegel[1,2] and Francesco Scotognella[1,2]*

Address: [1]Dipartimento di Fisica, Politecnico di Milano, Piazza Leonardo da Vinci 32, 20133 Milano, Italy and [2]Center for Nano Science and Technology@PoliMi, Istituto Italiano di Tecnologia, Via Giovanni Pascoli 70/3, 20133 Milano

Email: Francesco Scotognella - francesco.scotognella@polimi.it

* Corresponding Author

Transmission dependence on refractive index of the tunable filter for a Bragg mirror coupled to an ITO NC film.

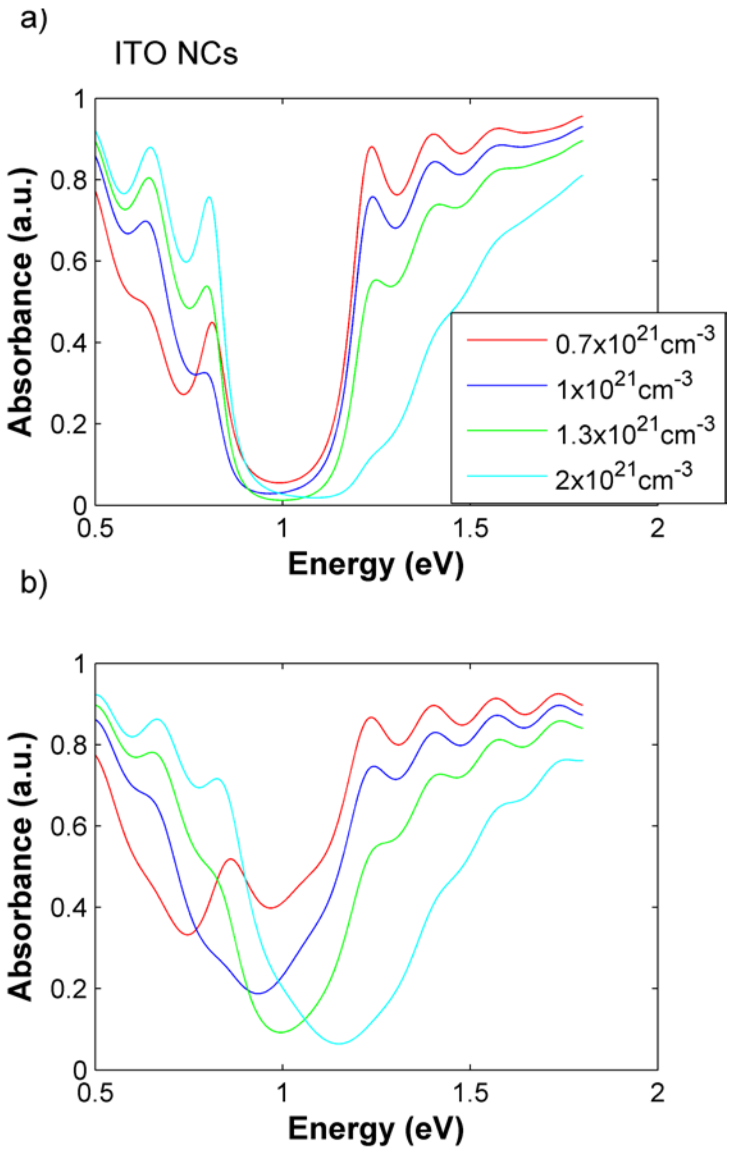

Figure S1: Transmission of the tunable light filter for a Bragg mirror coupled to an ITO NC film for varying carrier concentrations representing the band gap tuning of the Bragg mirror. In Figure S1a the refractive indexes of the two materials are 2 and 1.48, respectively, while in Figure S1b the refractive indexes of the two materials are 1.8 and 1.53, respectively.
